# Pseudo-endocrine Disorders: Recognition, Management, and Action

**DOI:** 10.1210/jendso/bvae226

**Published:** 2024-12-17

**Authors:** Michael T McDermott

**Affiliations:** Division of Endocrinology, Metabolism and Diabetes, University of Colorado Denver School of Medicine, Aurora, CO 80045, USA

**Keywords:** adrenal fatigue, Wilson's syndrome, reverse T3 syndrome, low-dose naltrexone

## Abstract

“Pseudo-endocrine disorders” refer to proposed conditions that have never been scientifically proven to exist but, due to widespread misinformation available on the internet and other media, are relatively commonly diagnosed and treated with equally unproven and sometimes dangerous treatments. Adrenal fatigue is a nonexistent condition that supposedly results from adrenal exhaustion and atrophy due to chronic stress and has been promoted as a potential explanation for a variety of symptoms. Testing consists of nonvalidated online surveys and salivary cortisol profiles while treatment is not evidence-based at best and can be dangerous. Wilson's syndrome and reverse T3 syndrome are also nonexistent conditions that supposedly result from impaired T4 to T3 conversion and competition of excess reverse T3 with T3 for T3 receptors. Testing involves measurement of axillary temperature and treatment consists of T3 therapy, often at very high and dangerous doses. Hypogonadism (“low T”) is frequently diagnosed in “men's health” clinics and other venues without actual hormone testing or further evaluation and is often treated with supraphysiologic testosterone therapy that suppresses endogenous gonadal testosterone and sperm production, leads to a lifelong need for testosterone therapy, and may have numerous other harmful effects. Low-dose naltrexone (LDN) therapy has been proposed as a treatment for multiple disorders including autoimmune conditions and other disorders resulting from aberrant immune mechanisms, but there is no valid evidence that LDN has any benefits. Management of patients with pseudo-endocrine disorders must involve careful listening, patient education, healthy lifestyle measures, and honesty, encouragement, and compassion.

The term “pseudo-endocrine disorder” does not have 1 clear and distinct definition [1]. The term could be used in reference to endocrine or metabolic disorders people believe they have because of misinformation they received from a health care provider, a personal trainer, or a family member or friend, and, despite previous normal test results, they request further unwarranted testing of their hormones or other indicators of endocrine function. It may apply to people who have read about endocrine disorders (real and unproven) in books or on the internet and may have even ordered hormone or metabolic testing online. It also refers to those who have been given “endocrine” diagnoses by providers based on symptoms alone without validated hormone testing. It also refers to people with endocrine or metabolic conditions that were diagnosed correctly (or not) by other providers and who are treated for these conditions with excessive hormone doses or with unproven, inappropriate, and even dangerous medications. Alternatively, an endocrine diagnosis may have erroneously been made as a result of lab assay error due to supplements and other conditions that adversely affect the accuracy of various tests [1]. Here the term is used mainly to describe proposed disorders that have never been scientifically proven to exist but that, due to widespread misinformation available on the internet and other media, are relatively commonly diagnosed and treated with equally unproven and sometimes dangerous treatments.

Pseudo-endocrine disorders are often diagnosed by nonendocrinologists, including people without a medical or health care degree, who promote non-evidence-based diagnoses and treatment suggestions from multiple unvalidated, non-evidence-based sources. People diagnosed with pseudo-endocrine disorders frequently later present to endocrinologists requesting further evaluation or treatment of these pseudo-disorders with diagnostic or treatment modalities that have no evidence for efficacy or safety and that may even be significantly harmful [1-5].

## Adrenal Fatigue

Proposed by James L. Wilson, a chiropractor and naturopath, in his 2001 book, *Adrenal Fatigue: The 21st Century Stress Syndrome*, adrenal fatigue is suggested to develop when the adrenal glands become exhausted due to chronic stress and are thus unable to produce adequate amounts of adrenal hormones to deal with the daily events and stresses of life [1]. It is said to occur in people who have chronic high stress (mental, emotional, or physical) in their work and/or family life. It is characterized by nonspecific symptoms such as fatigue, sleep disturbances, difficulty coping, body aches, digestive problems, and dependency on caffeine. Numerous practitioners and websites falsely promote this pseudo-endocrine condition. Adrenal fatigue was featured on *Dr. Oz* on October 22, 2015.

Affected people are encouraged to self-diagnose adrenal fatigue based on symptoms using scoring systems that are available on internet websites. Providers or patients themselves can also order a salivary cortisol profile in which multiple salivary cortisol samples are collected throughout the day and submitted to a lab for analysis (for a price); if the salivary cortisol levels fall below a normative line, the diagnosis of adrenal fatigue is said to be confirmed [1, 6].

Adrenal fatigue has never been scientifically proven to exist [1, 6-8]. Symptom scoring systems or salivary cortisol profiles have never been tested scientifically or validated as tools to evaluate the function of the hypothalamic-pituitary-adrenal (HPA) axis. Validated indicators of HPA function, such as serum cortisol and plasma ACTH levels and ACTH stimulation tests, are not part of the evaluation; when later done by conscientious diagnosticians, these validated HPA tests are usually normal unless the person has already been treated with steroid containing supplements. As clinician-scientists, we must be open to novel ideas and proposals. But rigorous verification by well-designed and well-conducted scientific investigations must still be the standard by which we evaluate and clinically apply new and innovative ideas. It is not sufficient, when patients’ health and well-being are concerned, to simply propose a hypothesis and apply it without diligent scientific investigation [1, 6-8].

Proposed treatments for adrenal fatigue start with a healthy, well-balanced diet, regular exercise, good sleep habits, stress reduction, and cessation of smoking and alcohol use [9]. No one can object to any of these measures; this is the same advice we give to almost all patients. But adrenal fatigue websites and promoters also suggest that patients use reflexology, take licorice root, or use a variety of supplements that are claimed to improve adrenal function. However, there are also recommendations for and links to purchase “real” or “raw” adrenal extracts that are made from bovine adrenal glands [10] and that contain actual steroid hormones in various amounts. The obvious potential for these products to cause secondary adrenal insufficiency is clearly a major concern. Once glucocorticoid-induced adrenal insufficiency has developed, tapering or discontinuing steroid therapy can result in glucocorticoid withdrawal syndrome [11, 12], significantly exacerbating the symptoms that initially prompted the use of these adrenal extracts.

The Endocrine Society has taken the lead in opposing misinformation about adrenal fatigue [7]. The website and literature provide clear warnings and guidance, such as “Adrenal fatigue is a diagnosis that patients are finding on the Internet or being given by alternative medicine practitioners, even though medical science recognizes no such condition”; “The fallacy of this logic is that there is no evidence that the stress of day-to-day life could have any such effect on the adrenals. Endocrinologists believe—correctly—that under stress your adrenals work harder and make more cortisol, not less” (Theodore C. Friedman, MD, PhD); and “Adrenal fatigue is not recognized by the Endocrine Society or any other endocrinology society, but adrenal insufficiency is.”

The Mayo Clinic website also has the following strong statements [8]: “The term often shows up in popular health books and on alternative medicine websites, but it isn't an accepted medical diagnosis” and “It's frustrating to have persistent symptoms your doctor can't readily explain. But accepting a medically unrecognized diagnosis from an unqualified practitioner could be worse. Unproven remedies for so-called adrenal fatigue may leave you feeling sicker, while the real cause—such as depression or fibromyalgia—continues to take its toll.”

Although serum cortisol testing ± the ACTH stimulation test are considered the gold standards for the diagnosis of true adrenal insufficiency, it is important to note that the use of steroid therapy or opioids [11-15] suppresses ACTH and cortisol production and produces reversible adrenal insufficiency; in my experience, steroid and opioid use is common among people with “adrenal fatigue.”

## Wilson's Syndrome

Proposed by E. Denis Wilson in 1990, Wilson's syndrome is suggested to be present when people develop reversibly impaired conversion of T4 to T3 as a prosurvival adaptation in response to chronic physical or emotional stress [1, 16]. Susceptible people include famine survivors or their descendants (Scotch, Irish, Russian, Native American); holocaust survivors or their descendants; survivors of divorce, death of a loved one, or stress (family or job); chronic dieters; yeast suffers; and people with hypoglycemia, eating disorders, and sleep disorders. The diagnosis is “made” by taking an axillary temperature with a mercury thermometer every 3 hours starting 3 hours after waking up; the body temperature readings are averaged over several normal days. If the mean body temperature is ≥ 1 degree below normal, the person “may” have Wilson's syndrome. The diagnosis does not involve testing TSH or thyroid hormone levels [1].

The suggested treatment for Wilson's syndrome involves cycling the body temperature up to normal with “proper T3 thyroid supplements” (doses up to 100 mcg daily) and then cycling down again. The cycle is repeated until the body temperature remains normal after stopping T3 supplements; this typically takes 3 to 4 cycles but may involve 11 to 12 cycles in difficult cases [1].

A 50-year-old woman died of an arrhythmia and heart attack while on amounts of thyroid hormone prescribed by Wilson; the Florida Board of Medicine accused him of “fleecing” patients with a “phony diagnosis.” The board and Wilson agreed to a 6-month suspension of his medical license, after which Wilson agreed to attend 100 hours of CME, submit to psychological testing, and pay a $10 000 fine.

The American Thyroid Association states emphatically on its website that there is no scientific evidence to support the existence of Wilson's syndrome and that the proposed treatment is unsafe and frankly dangerous [16].

## Reverse T3 Syndrome

Reverse T3 syndrome, which is similar to and an extension of Wilson's syndrome, was proposed in a 2016 book and website *Stop the Thyroid Madness* [17]. The proposal is that impaired T4 to T3 conversion not only reduces serum T3 levels but further that T4 is diverted into excessive amounts of reverse T3 (RT3) that then competes with T3 for T3 receptors in thyroid hormone-regulated tissues, further reducing peripheral tissue access to T3 action. Not only is there no scientific evidence to support the existence of this condition, but there is well-documented, published evidence demonstrating that T3 has 100-fold higher affinity for thyroid hormone receptors than does RT3; therefore excess RT3 is highly unlikely to impair T3 binding [18, 19]. The book also proposes ways to “flush reverse T3 out of the body.”

## Low-dose Naltrexone Therapy

The notion that endogenous opioids and opioid antagonists may play a role in healing and tissue repair led to the use of low-dose naltrexone (LDN) as a possible treatment for numerous disorders. Dr. Bernard Bihari, “a LDN Pioneer and Champion,” is featured in a 2002 online video describing his LDN work; an online tribute credits him with improving the lives and relieving symptoms in “tens of thousands (some say hundreds of thousands) of people with multiple sclerosis, rheumatoid arthritis, lupus, HIV/AIDS, autoimmune thyroid disease and even cancer.” In her book *Honest Medicine*, Julia Schopick proposes LDN as an “Effective, Time-Tested, Inexpensive Treatment for Life-Threatening Diseases” to include “multiple sclerosis, epilepsy, liver disease, lupus, rheumatoid arthritis and other disorders.”

A 2007 study published in the *American Journal of Gastroenterology* reported improvements in the Crohn's Disease Activity Index during 12 weeks of LDN treatment and for 4 weeks after stopping LDN in patients with active Crohn's disease [20]. Other studies followed with mixed results. However, a 2018 Cochrane Database Systematic Review [21] concluded there is “insufficient evidence to allow any firm conclusions regarding the efficacy and safety of LDN for patients with active Crohn's disease.” There is no valid evidence to support the use of LDN to treat other conditions, including autoimmune endocrine disorders [21].

## Pseudo-Hypogonadism in Males (“Low T”)

Low testosterone or “low T” clinics have sprung up in many communities. Testosterone levels are not always measured in these clinics since the diagnosis is often inferred by symptoms such as fatigue and low libido. When testosterone levels are actually measured and found to be low or low-normal, further testing to determine the cause of hypogonadism is usually omitted. Testosterone therapy is frequently recommended, sometimes in excessive amounts that result in frankly elevated serum testosterone levels that are known to be harmful. Failure to document true androgen deficiency before committing men to what may amount to lifetime testosterone replacement therapy is a serious disservice to these men. Furthermore, if hypogonadism is actually present, failure to investigate the cause is another serious disservice. Secondary hypogonadism is far more common than primary hypogonadism in men and is often due to other conditions such as opioid use, alcoholism, depression, sleep apnea, and various pituitary disorders, which are far more dangerous than the low testosterone levels they cause. Failure to diagnose these conditions also deprives men of the opportunity to resolve the hypogonadism by treating the underlying, and more serious, disorder. Finally, chronic testosterone therapy (warranted or not) suppresses a man's own hypothalamic-pituitary-gonadal axis and thereby suppresses endogenous testosterone and sperm production (the latter usually resulting in infertility); after prolonged therapy, testicular atrophy also commonly occurs. If a man without true hypogonadism is treated with testosterone replacement therapy and it is later stopped, full recovery of his hypothalamic-pituitary-gonadal axis may require up to 15 months [22]. Females treated with testosterone may develop hirsutism, acne, and insulin resistance; long-term safety in women has not been demonstrated [23].

## Pseudo-Cushing's Syndrome

Pseudo-Cushing's syndrome results from nonneoplastic conditions that activate the hypothalamic-pituitary-adrenal axis and actually produce hypercortisolism and Cushingoid features [24]. These conditions include alcoholism, depression, and poorly controlled diabetes. The usual screening tests for Cushing's syndrome (24-hour urine free cortisol, 1 mg overnight dexamethasone suppression test, bedtime salivary cortisol) can all be positive in people with these conditions. However, adequate treatment of these disorders usually results in the resolution of hypercortisolism. Morbid obesity can also have many features suggestive of Cushing syndrome. But selective central obesity, proximal muscle weakness, bruising, and violaceous striae are usually not present with morbid obesity and 24-hour urine free cortisol excretion is usually normal.

High estrogen levels from oral contraceptive pills and in pregnancy promote increased hepatic production of cortisol binding globulin, which binds cortisol for transport in the circulation. Serum total cortisol levels are increased approximately 67% in women who take oral contraceptive pills; pregnancy causes similar elevations. This can result in false-positive testing for both baseline and dexamethasone-suppressed serum cortisol levels [25]. Tests that measure free cortisol (urinary free cortisol, salivary free cortisol) are not affected by oral contraceptive pills or pregnancy.

## Pseudo-Pheochromocytoma

The term “pseudo-pheochromocytoma” has been applied to symptom complexes consisting of palpitations, headaches, anxiety, tremors, and sometimes hypertension that are similar to those caused by pheochromocytomas, but with completely normal serum and urine metanephrine and catecholamine levels and that are due to other conditions. Treatment of these patients is reassurance [26]. The term “pseudo-pheochromocytoma” also applies to 2 situations in which catecholamines and/or metabolites are actually elevated. Urinary metanephrine and normetanephrine levels in hospitalized patients (intensive care unit and non-intensive care unit) can be very elevated and overlap significantly with those seen in pheochromocytomas and paragangliomas [27-30]. Therefore, elevated catecholamines and metabolites should be interpreted with caution in the setting of severe illness or hospitalization and are best repeated at a later date after discharge and recovery. Also, the use of levodopa/carbidopa for Parkinson's disease significantly elevates plasma and urine dopamine levels without affecting norepinephrine or epinephrine levels [31, 32].

## Hashimoto Encephalopathy

Hashimoto encephalopathy is a misnomer referring to an acute encephalopathy of unknown cause that typically presents with impaired mental status, somnolence, multiple stroke-like episodes, and seizures [33, 34]. It was previously termed “Hashimoto encephalopathy” because many, but not all, affected patients were found to have positive antithyroid antibodies in the serum and cerebrospinal fluid. However, the encephalopathy does not appear to be related to thyroid antibodies or thyroid dysfunction and responds best to steroid therapy. Therefore, use of the term “Hashimoto encephalopathy” is discouraged, and the condition has become more accurately referred to as “steroid responsive encephalopathy associated with autoimmune thyroid disease” [33, 34].

## Biotin Interference with Hormone Assays

Biotin is a reagent component for many hormone assays. High-dose biotin supplements (5000-20 000 mcg/day for hair loss and brittle nails) can cause falsely abnormal hormone measurements during biotin ingestion. High biotin concentrations can falsely elevate levels in biotin-streptavidin competitive immunoassays (free T4, free T3, TRAb, cortisol, testosterone, estradiol) and falsely lower levels in biotin-streptavidin immunometric assays (TSH, PTH, ACTH, LH, FSH) [35, 36]. Graves’ disease can thus be erroneously diagnosed (low TSH, high free T4 and T3, positive TRAb) in someone taking high-dose biotin. An 8-hour abstinence from biotin often corrects this anomaly, but most authorities recommend ≥ 3 days of abstinence. The amount of biotin in most multivitamins is not sufficient to cause assay interference. Other hormones, like ACTH and PTH, may show falsely low values if blood samples are not collected properly (for example in the correct tube and placed on ice) resulting in hormone decay.

## Patient-Centered Approach to Patients Who Present With a Pseudo-endocrine Diagnosis

All providers will develop their own personalized approach that works best for their patients, but I find it best to always remember three crucial points: (1) the patient is not to blame for their diagnosis; like all others, they deserve our full respect, attention, and compassion; (2) the patient's quality of life is poor and they are frustrated; (3) it is an honor that they entrust me with an opportunity to help them achieve better health and quality of life.

I listen attentively, perform a good physical examination, and offer additional testing if appropriate. For example, validated tests of the hypothalamic-pituitary-adrenal axis (cortisol, ACTH, cosyntropin stimulation testing, 21 hydroxylase antibodies) can be considered for those falsely diagnosed with adrenal fatigue and the results fully explained to the patient with reassurance that the tests show no evidence of adrenal disease or dysfunction. For men who may have been falsely diagnosed with “low T,” appropriate testing protocols to document testosterone deficiency (fasting Am serum testosterone levels on 2 separate days) should be offered. If androgen deficiency is verified, gonadotropin testing should be done to determine if the hypogonadism is primary or central. The cause of central hypogonadism (pituitary disease, hemochromatosis, hyperprolactinemia, thyroid disease, sleep apnea, depression, alcohol abuse, opioid use, obesity, chronic illness) or primary hypogonadism (drugs, radiation therapy, trauma, infection, genetics) should then be thoroughly evaluated so that the patient can be informed of the cause and expected course of the condition.

I also explore nonendocrine and psychiatric contributors to their symptoms. I understand and admit that current testing options do have some limitations. I recommend good nutrition, regular exercise, good sleep habits, and stress reduction for all patients. And I always provide honesty, encouragement, and compassion [1, 9].

## Public Action

Over the past 30 years, we have seen a disturbing proliferation of print, broadcast, and internet claims and advertisements concerning fabricated diseases that have no actual scientific or credible clinical evidence for their existence and for which unproven and potentially harmful remedies are openly shilled for profit. We have seen practitioners proclaim themselves to be experts in hormonal therapy without any formal training and confidently promote hormone treatments without adequate endocrine evaluations. We have heard practitioners make astonishing promises regarding the benefits of herbal, supplemental, and other unproven therapies that they themselves sell in their offices and/or online. We have witnessed people being given harmful and even dangerous products that contain animal whole organ (most commonly thyroid and/or adrenal) extracts or hormonal injections that produce highly elevated levels of sex hormones (especially testosterone) without any concern for short-term safety or long-term outcomes. And we have heard about the surprisingly high costs incurred by people who visited these practitioners and had no benefits or even had frankly concerning results, despite being promised symptom improvement, safety, and full insurance coverage [1-5].

What is our responsibility as concerned practitioners and citizens in the face of these rogue practitioners and practices? We clearly have a primary obligation to protect each of our patients and communities from these unscrupulous, charlatan practitioners and the unproven, costly, and sometimes dangerous treatments they promote. We can do this through one-on-one in-person education with our patients. We can also direct them to credible professional websites such as those of the Hormone Foundation (Endocrine Society), American Thyroid Association, and Mayo Clinic (see reference list).

More broadly, however, we should seek opportunities to bring these issues to the attention of the general public. For example, I was interviewed on a local television station about testosterone therapy. During this brief interview, I informed listeners that low testosterone levels are often a manifestation of more serious underlying medical conditions or of medications, that treatment of these conditions should be the primary focus of therapy instead of jumping immediately to testosterone therapy, and that low testosterone levels caused by these conditions are often restored to normal, without a need for lifelong testosterone replacement therapy, if the underlying medical conditions are appropriately managed. As physicians, we have much more influence over these issues than anyone else in our communities. The public listens to physicians; what we say makes a difference.

We can encourage our local and national professional organizations (state medical societies, American Medical Association, American College of Physicians, the Endocrine Society, the American Association of Clinical Endocrinologists, and the American Thyroid Association) to publish fact sheets, official statements, and guidelines regarding these types of practices. And we can write editorials and commentaries in our local newspapers. One particularly well-written piece by Dr. Lisa Pryor was published in *The New York Times* (January 5, 2018) and was titled “How to Counter the Circus of Pseudoscience” [5].

Another option is to report rogue practitioners and practices to your local state medical board. A small group of Colorado physicians reported a local physician who was treating patients who complained of fatigue with a combination of high-dose prednisone and desiccated thyroid extract without testing for either adrenal insufficiency or hypothyroidism prior to initiating this aggressive therapy. I was asked to testify in court regarding this case. The outcome was removal of his medical license; he subsequently retired.

## Summary and Conclusions

We all have experienced evaluating and managing patients who have symptoms that are suggestive of an endocrine disorder but that are very nonspecific and could be due to a variety of other causes. When endocrine testing results are normal, many of these people seek advice elsewhere, and some are given diagnoses by physicians, nonphysicians, naturopaths, friends, health club members, and websites that are not supported by proper lab tests or that are not actually validated conditions. Many of these pseudo-conditions were created by people who have no respect or regard for evidence-based medicine or for the welfare of patients who are duped into believing in these false diagnoses and non-evidence-based remedies.

Some of the common pseudo-endocrine conditions are adrenal fatigue, Wilson's low T3 syndrome, reverse T3 syndrome, and “low T.” Common remedies that are most likely not harmful and are potentially beneficial include diet, exercise, vitamins, minerals, herbs, some supplements, better sleep, low-dose naltrexone, and stress reduction. But some proposed treatments are harmful and potentially dangerous, including animal whole gland adrenal extracts, high-dose liothyronine, and testosterone pellets.

These pseudo-conditions vex legitimate healthcare providers because the people have been led to believe that they have real diseases with valid and beneficial treatments; however, these are fake diagnoses that are not supported by actual science and that are proposed to be responsive to non-evidence-based treatments. More importantly, they actually do harm by making people believe they have found the answer to their symptoms and can stop pursuing a true answer. Furthermore, these pseudo-diagnoses often lead to inappropriate and harmful treatments. For example, some people with “adrenal fatigue” are treated with animal adrenal extracts that contain intact steroid hormones that can do significant harm by causing secondary adrenal insufficiency, which is well known to impair quality of life and can be life-threatening. An erroneous diagnosis of hypogonadism or “low T” may lead to lifelong testosterone replacement therapy that actually results in secondary (hypogonadotropic) hypogonadism, impaired sperm production, and infertility. Testosterone pellets may be especially harmful because they often produce highly supraphysiologic serum testosterone levels that last as long as the pellets function. While physiologic testosterone replacement in men with true hypogonadism has been shown to be safe and beneficial, safety has not been demonstrated with supraphysiologic testosterone replacement, and this raises the specter of promotion of cardiovascular disease and neoplasia. The only individuals who benefit from these bogus therapies are those who profit from the sale of nonvalidated diagnostic kits and/or the fake treatments.

I believe it is critical to not blame people for having these diagnoses. They unwittingly trusted unscrupulous providers, literature, or websites that provided them with false information and non-evidence-based recommendations. People with symptoms simply want to feel better. These people have been taken advantage of. All patients are important and should not be treated this way.

I consider it an honor when a person entrusts their health and healthcare to me. I give them my utmost attention and the full measure of my respect and compassion. I listen carefully to their complaints and symptoms, take a complete history, and do a good physical examination. I review their previous lab results and explain which ones are valid and which ones are not and offer additional testing if appropriate. I explain that it can be very difficult to pinpoint a single cause for many nonspecific symptoms but that we can make progress if we move forward with a positive attitude and healthy lifestyle measures such as good nutrition, regular exercise, good sleep habits, and stress reduction. Treatment of any identified medical conditions should be undertaken with the best evidence-based treatment approaches. I recommend that people come back within a reasonable time to evaluate how they are doing with our treatment plan. This approach will not work for everyone but will benefit a substantial number of people. It is very gratifying for me to hear people tell me “thank you,” not because I cured them but because I listened to them, respected them, and tried to help them.

## Data Availability

There is no actual research data in this manuscript. It is primarily a review article mixed with personal observations and experience.
